# Genetic Diversity and Population Structure Analysis of *Dalbergia Odorifera* Germplasm and Development of a Core Collection Using Microsatellite Markers

**DOI:** 10.3390/genes10040281

**Published:** 2019-04-06

**Authors:** Fu-Mei Liu, Ning-Nan Zhang, Xiao-Jin Liu, Zeng-Jiang Yang, Hong-Yan Jia, Da-Ping Xu

**Affiliations:** 1 Research Institute of Tropical Forestry, Chinese Academy of Forestry, Longdong, Guangzhou 510520, China; liufumei1115@163.com (F.-M.L.); ningnanzhang@126.com (N.-N.Z.); xjliucaf@163.com (X.-J.L.); yzengjiang@126.com (Z.-J.Y.); 2 The Experimental Centre of Tropical Forestry, Chinese Academy of Forestry, Pingxiang 532600, China; rlzxjhy@163.com; 3 Guangxi Youyiguan Forest Ecosystem Research Station, Pingxiang 532600, China

**Keywords:** *Dalbergia odorifera* T. Chen, genetic diversity, core collection, SSR marker, Rosewood, conservation

## Abstract

*Dalbergia odorifera* T. Chen (Fabaceae) is a woody tree species indigenous to Hainan Island in China. Due to its high medicinal and commercial value, this tree species has been planted over 3500 ha^2^ in southern China. There is an urgent need for improvement of the *D. odorifera* germplasm, however, limited information on germplasm collection, conservation, and assessment of genetic resources is available. Therefore, we have built a database of 251 individuals collected across the whole of southern China, which included 42 wild trees and 210 cultivated trees, with the following objectives. (1) Evaluate genetic diversity and population structure of the database using 19 microsatellite markers and (2) develop a core collection for improvement and breeding programs. Totally, the 19 microsatellite markers harbored 77 alleles across the database with the polymorphic information content (PIC) ranging from 0.03 to 0.66. Medium genetic diversity level was inferred by Nei’s gene diversity (0.38), Shannon’s information index (0.65), and observed (0.33) and expected heterozygosity (0.38). Structure analysis showed that four was the optimum cluster size using the model-based Bayesian procedure, and the 251 *D. odorifera* individuals were grouped into five populations including four pure ones (RP1-4) and one mixed one (MIX) based on their maximum membership coefficients. Among these populations, the expected heterozygosity varied from 0.30 (RP3) to 0.38 (RP4). Analysis of molecular variance (AMOVA) showed 11% genetic variation existed among populations, and moderate population differentiation was inferred by the matrix of pairwise Fst (genetic differentiation among populations), which was in the range of 0.031 to 0.095. Moreover, a core collection of 31 *D. odorifera* individuals including six wild and 25 cultivated trees was developed, which was only 12.4% of the database but conserved the whole genetic diversity. The results of this study provided additional insight into the genetic structure of the large *D. odorifera* germplasm, and the core collection will be useful for the efficient and sustainable utilization of genetic resources, as well as efficient improvement in breeding programs.

## 1. Introduction

*Dalbergia odorifera* T. Chen, formerly named *Dalbergia hainanensis* Merr. et Chun, is a semideciduous perennial woody tree species of high medicinal and commercial value. The heartwood of its root and stem is a traditional Chinese medicine, named “Jiangxiang”, which plays crucial roles in cardiovascular treatments [1], diabetes [2], blood disorders, etc. [3,4] as it contains sets of complex and useful chemical components [5,6,7,8,9]. The heartwood of this species, also named “Hualimu” or “Huanghuli” (Chinese name), is a precious fragrant rosewood with extremely high value in the luxury furniture and craft markets in China. Often the most highly valued timber trees are the most threatened species in their native habitats [10]. For example, *D. odorifera*—an indigenous to Hainan Island in southern China and is confined to a relatively narrow tropical geographic area [11]—that has been overexploited for a long time and has been listed on the IUCN (International Union for Conservation of Nature’s) red list by World Conservation Monitoring Centre (WCMC) since 1998 [12]. The resource has also been promoted to a second-grade state protected species by the Chinese government. It is now in danger of extinction resulting from illegal logging. Currently, only a limited number of plants exist in the remaining forest fragments [11,13].

Due to its high medicinal and commercial value, *D. odorifera* was introduced to subtropical areas of Guangdong, Guangxi, and Fujian provinces in China in the 1950s. After several decades, the introduced trees exhibited a satisfactory growth performance, and, at most sites, has even formed valuable heartwoods, which take approximately 50 years to mature in the wild [14]. Recently, more and more farmers planted *D. odorifera* trees because of its ecological and economic importance. Moreover, the plantation area of this species has exceeded 3500 ha^2^ in southern China. There is an urgent need for improvements of the *D. odorifera* germplasm, particularly for higher yield of heartwood in a shorter harvest cycle. Such improvements are based on the germplasm collection, conservation, and assessment of genetic resources.

Germplasm is the basis of plant breeding programs [15]. During the last several decades, there has been remarkable progress in *D. odorifera* germplasm collection [16,17,18]. Eventually, a dataset of 251 individuals has been collected and conserved in the Research Institute of Tropical Forestry (Chinese Academy of Forest, CAF) and in Guangxi Youyiguan Forest Ecosystem Research Station at the Experimental Center of Tropical Forestry (CAF). These individuals came from all over southern China, covering its native habitat (Hainan Island) and introduced sites (Guangdong, Guangxi, Hunan, and Fujian provinces). However, the original information on these introduced germplasm is confusing due to frequently disordered introductions within and among different non-native regions. Little is known about the genetic diversity and structure of the whole collection. These issues have limited the efficient and sustainable utilization of the genetic resources. Hence, a core collection has been proposed to deal with this issue.

A core collection is a subset of the whole germplasm collection, and aims to create a uniform representation of the original genetic space with equal weights across this space [19]. A good core collection is one that has no redundant accessions, is small enough to be easily managed, and represents the total genetic diversity [20]. Notably, core collections have been developed for many tree species, including apple (*Malus domestica* Borkh) [21,22], avocado (*Persea americana* Mill.) [23], and chestnut (*Castanea sativa* Mill.) [24]. However, no core collection has been developed for *D. odorifera* yet. Additionally, information on the genetic diversity and genetic variation of plant species has been well demonstrated using molecular markers [25,26,27,28], which are essential to core collection, germplasm identification, and breeding purposes [29]. Therefore, we conducted a comprehensive survey on genetic diversity and genetic variation for the database of 251 *D. odorifera* individuals using a set of 19 microsatellite markers, and developed a core collection using PowerCore software [30]. The main objectives of this study were to (1) evaluate genetic diversity and population structure of the *D. odorifera* germplasm and (2) develop a core collection conserving the entire diversity for improvement and breeding programs. The findings of this study will provide a useful resource as well as guidance for better germplasm utilization in genetic improvement, and serve as a database for identification, parentage, and traceability purposes.

## 2. Materials and Methods

### 2.1. Plant Materials and DNA Extraction

A total of 251 individuals, including 42 wild trees and 209 cultivated trees, was collected from the native habitat and introduced sites of *D. odorifera* across the southern China (Table S1). Among the cultivated individuals, 62 came from Hainan Island, 69 from Guangxi province, 52 from Guangdong province, 20 from Fujian province, and 6 from Hunan province. Permission for leaf sample collection was obtained from the local managers. Ten young leaves were collected from each individual and sealed in plastic bags with desiccants. Total genomic DNA was extracted for each sample using the Hi-DNAsecure Plant Kit (Tiangen, Beijing, China) according to the manufacturer’s instructions. The quality and quantity of DNAs were determined by NanoDrop 2000 (Thermo Scientific, Wilmington, DE, USA).

### 2.2. PCR and Capillary Electrophoresis

A set of 19 microsatellite markers was developed in our previous study [13] and used in the present study (Table S2). Subsequently, PCR reactions with the designed primers [13] were carried out using DNAs for all the 251 samples. PCR reactions were of 15 µL final volume, containing 10.25 µL water, 1.5 µL 10×  DNA polymerase buffer, 1.5 µL MgCl_2_ (25 mM), 0.3 µl dNTPs (10 mM each), 0.15 µL of each primer at 10 µM, 0.3 µL Taq polymerase at 5 units/ µL (TaqUBA), and 1 µL of genomic DNA (40–50 ng). Then, 35 cycles of 94 °C for 15 s, appropriate annealing temperature for 15 s, and 72 °C for 30 s were performed, following the predenaturation at 94 °C for 3 min. All the PCR reactions were repeated at least once. Their diluted PCR products were mixed with 12.5 Hi-Di formamide and 0.25 µL of size standard (Shanghai Generay Biotech Co., Ltd, Shanhai, China), and were then separated by capillary electrophoresis and genotyped with an ABI 3730 Genetic Analyzer (Applied Biosystem, Foster, CA, USA) at Shanghai Generay Biotech Co., Ltd., Shanghai, China. Peak identification and fragment sizing were done using Gene Mapper v4.0 (Applied Biosystems) with the default settings.

### 2.3. Population Structure

The genetic structure of the investigated database was analyzed using STRUCTURE 2.0 [31]. The number of discontinuous K was estimated from one to ten with twenty iterations. Both the length of burn-in period and value of MCMC (Markov chain Monte Carlo) were set to 100,000 times [32]. The admixture model was used with correlated allele frequencies, the options “popinfo” and “popflag” were both set to zero to consider that the sampled individuals were of unidentified origin. Next, the optimum value of cluster (K) was harvested online according to the highest lnP(D)-derived Δ K (log probability of data derived delta K) value [33]. Repeated sampling analysis and the genetic structural plot were performed by CLUMPAK [34]. In this approach, each individual was assigned to populations (named RP1-RP(K) and MIX) based on its maximum membership coefficient using a threshold value of 0.65 for the Q statistic according to the optimum K value. Each individual was assigned to RPs when the maximum membership coefficient of the individual above 0.65, otherwise, it was classified into MIX [35]. Unweighted neighbor-joining phylogenetic trees and principal coordinate analyses were both performed based on the dissimilarity matrix calculated with Manhattan index, using DARwin software (version 6.0.9) [36,37]. To summarize the patterns of variation in multilocus dataset, and principal coordinate analysis (PCoA) was also performed using GenAlEx version 6.5 software based on the matrix of pairwise Nei’s genetic distance [38].

### 2.4. Genetic Diversity Statistics

The frequency of null alleles (FNA) and scoring errors were estimated using the Microchecker software 2.2.3 [39]. Genetic diversity parameters such as allele frequency, observed number of alleles (Na), effective number of alleles (Ne), expected (He) and observed heterozygosities (Ho), Nei’s gene diversity (GD), the percentage of polymorphic loci (PPB), gene flow (Nm), the Shannon’s information index (I), and Wright’s fixation index (F) were calculated using POPGENE v1.3.1 software [40]. The polymorphism information content (PIC) was calculated for each locus using the online program PICcalc [41]. F-statistics, including inbreeding coefficient within individuals (F_IS_), genetic differentiation among populations (F_ST_), and the pairwise Fst, were computed using GenAlEx version 6.5, which was also conducted the hierarchical analyses of molecular variance (AMOVA) [38]. The Ewens–Watterson test for neutrality at each locus was performed using POPGENE v1.3.1 [40].

### 2.5. Construction and Evaluation of the Core Collection

A core collection—a reduced number of samples which represents the greatest diversity of the initial collection—was generated using PowerCore (v. 1.0) with a heuristic search [30]. The representativeness of the core collection was validated according to the following criteria [22,24,42]. (1) Harboring all alleles present in the entire collection; (2) no significant differences in variability parameters (Ho and He) between the two collections, all the comparison were carried out with SPSS v.16.0 (SPSS, Chicago, IL, USA) at significance level below 0.05 (*p* < 0.05); (3) validating the core collection with unweighted neighbor-joining dissimilarity trees using DARwin software (version 6.0.9) [36]; (4) and a matrix of pairwise Nei’s unbiased genetic distance [43] was constructed using POPGENE v1.3.1 software [40], based on which, an Unweighted Pair Group Method with Arithmetic Mean (UPGMA) tree was constructed to reveal the relationship among individuals in the collection using NTSYS-pc software (version 2.1) [44].

## 3. Results

### 3.1. Polymorphism of 19 Microsatellite Markers

Nineteen microsatellite markers were used in the present study (Table S2), a total of 77 alleles were detected across the 251 *D. odorifera* individuals, and the number of alleles detected per locus varied from two to seven (Table 1). The polymorphic information contents (PIC) ranged from 0.03, at S26, to 0.66, at S21, with a mean of 0.32. The mean Shannon’s Information index (I), observed (Ho), and expected heterozygosity (He) were 0.65, 0.33, and 0.38, respectively. Furthermore, null alleles were found at loci S03, S04, S09, S22, S23, S24, S26, and S28. All 19 microsatellite loci were selectively neutral according to the Ewens–Watterson test for neutrality (Table S3).

### 3.2. Population Structure of D. odorifera Germplasm

**Figure 1:**
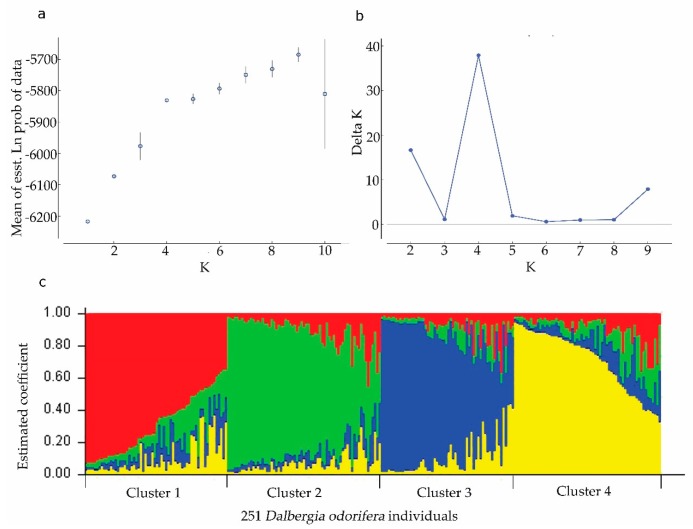
Results of STRUCTURE analysis for 251 *Dalbergia odorifera* individuals based on microsatellite data. (**a**) Estimation of population using mean of estimated lnP(D) (log probability of data) with cluster number (K) ranged from one to ten. (**b**) Estimation of population using lnP(D)-derived delta K with cluster number (K) ranged from one to ten. (**c**) Four estimated clusters of the 251 *D. odorifera* individuals presented with different colors inferred by STRUCTURE analysis. Clusters 1–4 were presented by red, green, blue, and yellow, respectively. Each bar represented an individual, in which, different color represents the estimated membership coefficients using the Q statistic.

**Figure 2:**
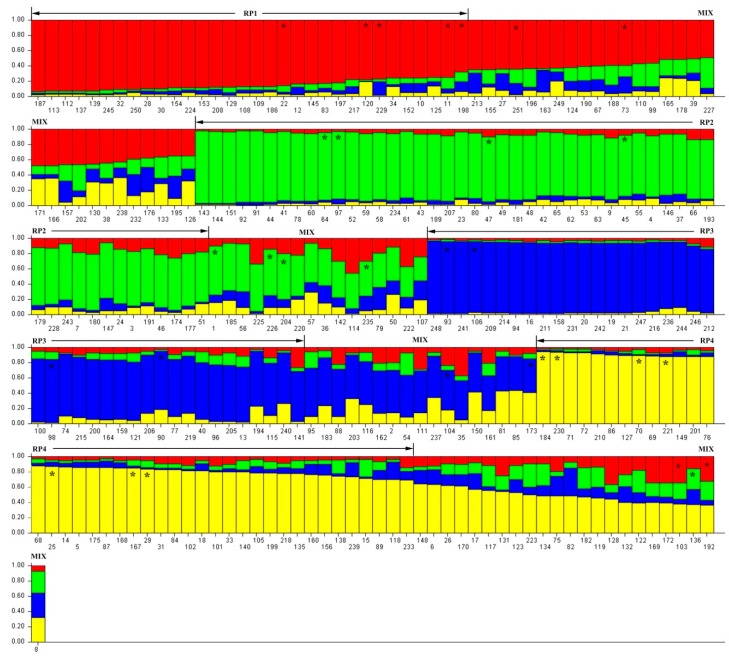
Details of hierarchal structure analysis on 251 *Dalbergia odorifera* individuals. RP1, RP2, RP3, RP4, and MIX: population code; *: core collection member; Arabic numerals: ID for each individual, see Table S1.

An admixture model-based approach was implemented to investigate the population structure of 251 *D. odorifera* individuals. The optimum cluster was four, which was generated from the STRUCTURE HARVESTER website with the largest lnP(D)-derived Δ K (log probability of data derived delta K) value (Figure 1a–c). Subsequently, the 251 individuals were classified into five populations based on their maximum membership coefficients, which were designated as RP1 to RP4, and a mixed population MIX (Figure 2). Among these populations (Table 2), MIX contained the largest amount of member 86 including 13 wild ones, followed by RP2 (51, three wild), RP3 (41, 15 wild), RP4 (41, six wild), and RP1 (33, five wild). The information on the geographic origins, types and inferred reconstructed populations is available in Table S1.

**Figure 3:**
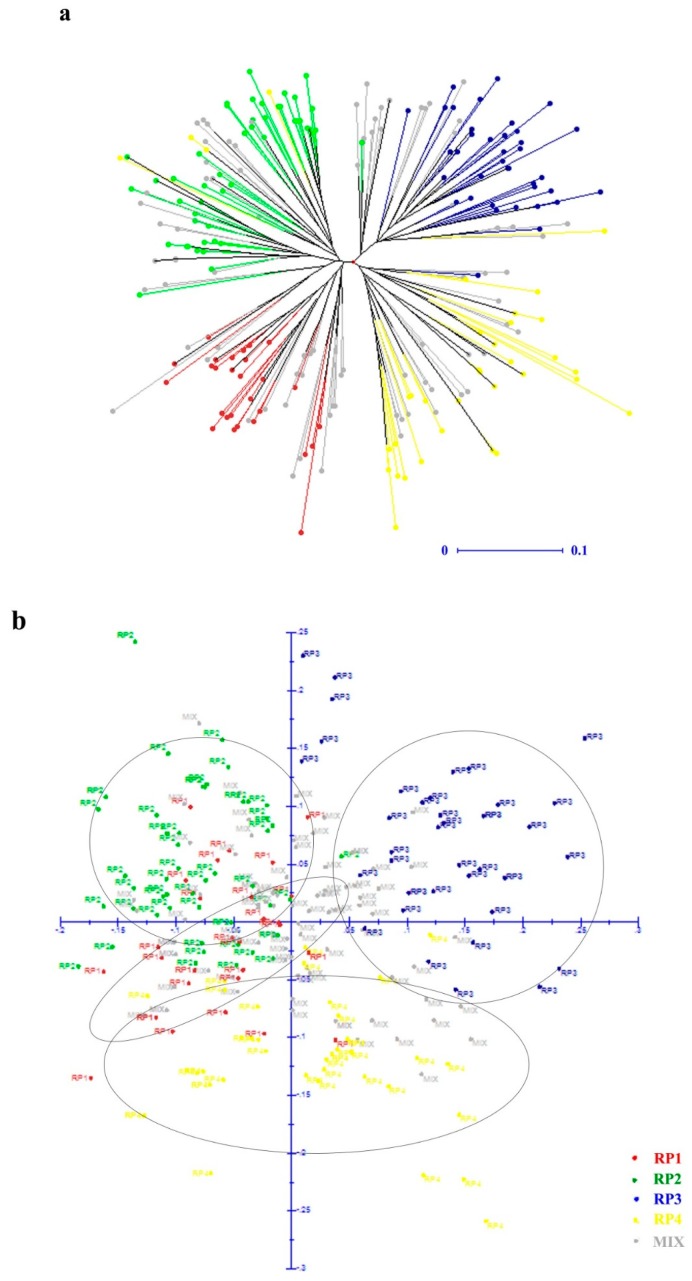
Relationships of the 251 *Dalbergia odorifera* individuals. (**a**) An unweighted NJ (neighbor-joining) tree based on the dissimilarity matrix calculated with Manhattan index among 251 *D. odorifera* individuals. (**b**) Principal coordinate analysis (PCA) based on the dissimilarity matrix, the x-axis, the first principal coordinate explained 9.67% of variation; the y-axis, the second principal coordinate explained 8.20% of variation.

Neighbor-joining (NJ) phylogenetic analysis and principal component analysis (PCA) were used to detect the genetic relationship across the 251 individuals based on the dissimilarity matrix calculated with Manhattan index. Four clusters were clearly distinguished by both the NJ dendrogram tree and the PCA plot (Figure 3). Moreover, the x- and y-axis in the PCA plot explained 9.67% and 8.20% of variance within the molecular data, respectively.

### 3.3. Genetic Diversity and Variation of D. odorifera Germplasm

Among the five populations (Figure 2, Table S1), the number of polymorphic loci varied from 17 (RP3) to 19 (MIX and RP1), along with the percentage of polymorphic loci (PPB) from 89.47% to 100.00% (Table 2). The largest values of allele (total number of detected alleles), Na (observed mean number of alleles), and Np (number of private alleles) were all detected in MIX, which were 66, 1.74, and 9, respectively. Moreover, RP4 presented the highest genetic diversity among these populations, showing the largest value of expected heterozygosity (He) 0.38 and Nei’s gene diversity (GD) 0.37. Additionally, both the expected heterozygosity and Nei’s gene diversity was 0.38 within the 251 *D. odorifera* individuals.

Both analysis of molecular variance (AMOVA) and pairwise Fst analysis were performed to investigate the genetic variations among these populations. The results showed that 11% of the total genetic variation occurred among populations (Table 3). Moderate genetic differentiation was indicated by pairwise Fst ranging from 0.031 to 0.095 (Table 4). The highest level appeared between RP2 and RP3, whereas the lowest appeared between RP2 and MIX. Furthermore, the principal coordinate analysis (PCoA) was carried out using the GenAlEx version 6.5 based on the matrix of pairwise Nei’s unbiased genetic distance. The results showed that 40.11% of the variance within the molecular data was illustrated by the first axis, and 32.14% explained by the second axis (Figure S1). Additionally, the five populations could clearly group into three clusters: MIX, RP2, and RP1 represented one cluster, while RP3 and RP4 each represented a cluster.

### 3.4. Core Collection Development of Dalbergia odorifera

**Figure 4:**
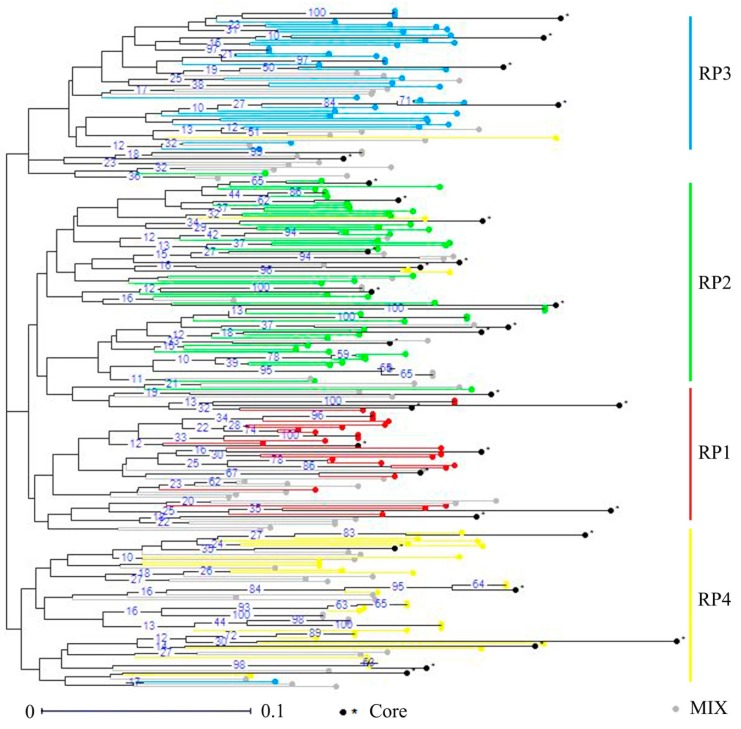
Distribution of the core collection in the NJ tree of the whole database based on genetic dissimilarity.

**Figure 5:**
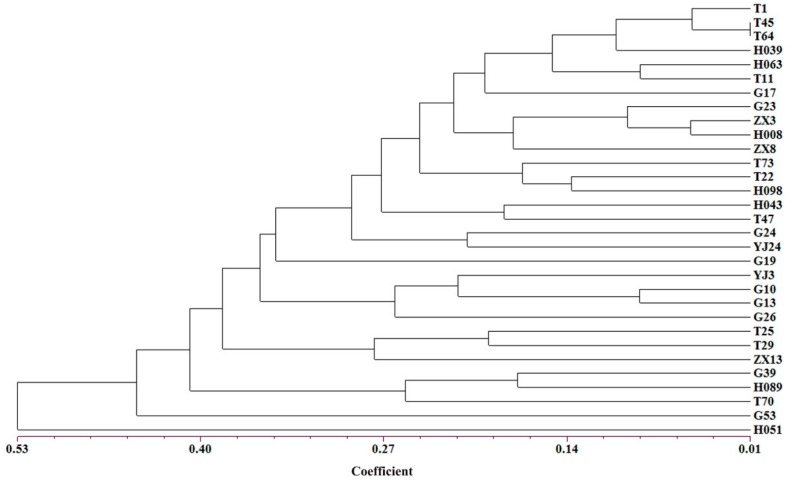
The Unweighted Pair Group Method with Arithmetic Mean (UPGMA) tree of the core collection based on Nei’s unbiased genetic distance.

To conserve an overview for the whole genetic diversity of the germplasm, a core collection that contained 12.4% of the 251 *D. odorifera* individuals was constructed using the PowerCore software with the advanced M-strategy (Table S1). The core collection included 24 cultivated trees and seven wild trees, and harbored a total of 77 alleles with the observed allele number varying from two to seven per locus (Table S4). The observed number of alleles was 4.05, which was exactly the same as the whole database. Moreover, there was no significant difference on genetic diversity indices between the core collection and the whole database, and the observed and expected heterozygosity was 0.33 and 0.44, respectively (Table 5). Details on genetic diversity and variations statistics of the core collection were available in Table S5 and Table S6. Moreover, the NJ dendrogram tree showed that the core collection was uniformly distributed in the entire *D. odorifera* germplasm based on genetic dissimilarity (Figure 4), and based on the matrix of unbiased Nei’s genetic distance, the phylogenetic relationships among the 31 individuals was exhibited in the UPGMA tree (Figure 5).

## 4. Discussion

### 4.1. Genetic Diversity and Population Structure of D. odorifera Germplasm

Genetic diversity plays an important role in genetic improvement through breeding programs [45]. However, information on *D. odorifera* genetic diversity is limited. Prior to the present study, only two reports have been available: Yang et al. [11] evaluated genetic diversity of 77 wild *D. odorifera* trees using six RAPD (random amplified polymorphic DNA) markers and Liu et al. [45] assessed 42 wild trees using 19 SSR (simple sequence repeat) markers. Both reports indicated medium genetic diversity level, which was inferred by Nei’s gene diversity value of 0.21 (RAPD) and 0.36 (SSR). Compared to the two studies, a relatively higher genetic diversity level with a higher Nei’s gene diversity value 0.38 (Table 1) was exhibited in our result on assessing a dataset of 251 *D. odorifera* individuals using 19 microsatellite markers. The difference in genetic diversity may be mainly resulted from the larger population size investigated in the present study [20,46], or alternatively, from the different numbers [47] or types of molecular markers [48] used in these studies.

Assessment on the genetic diversity and population structure of a species is essential to evaluate the applicable potential of a new germplasm resource, and the prior knowledge of genetic diversity and pairwise relatedness can provide beneficial clues for efficient utilization in large collections of genetic resources [35,49,50]. Therefore in this study, we assessed the genetic diversity of 251 *D. odorifera* individuals collected from its whole native habitat and introduced sites covering five provinces in southern China. In total, 19 microsatellite markers harbored 77 alleles across the whole database with the mean polymorphic information content (PIC) of 0.32 (Table 1). Medium genetic diversity level of *D. odorifera* was inferred by Shannon’s information index and observed and expected heterozygosity of 0.65, 0.33, and 0.38, respectively (Table 1). The results of genetic diversity indices were comparable to *Dalbergia sissoo* Roxb. (PIC 0.30) [51], but much lower than these reported for other Dalbergia species, such as *Dalbergia monticola* Bosser & R. Rabev. (He 0.83) [52], *Dalbergia nigra* (Vell.) Benth. (Ho 0.69, He 0.75) [53], *Dalbergia cochinchinensis* Pierre ex Laness (Ho 0.56, He 0.55), and *Dalbergia oliveri* Prain (Ho 0.76, He 0.73) [54]. This difference may be attributed to that *D. odorifera* is an endemic tree species with original distribution restricted to the small regions of Hainan Island, which is concordant with the general trend that distribution-restricted plant species are associated with relatively low genetic diversity [55,56,57,58].

The Bayesian model-based structure analysis is widely used for the inference of hidden population structure in plant species [32]. In this study, structure analysis showed that four was the optimum cluster for the 251 *D. odorifera* individuals (Figure 1). Both neighbor-joining (NJ) phylogenetic analysis and principal component analysis (PCA) verified the structural pattern as distinctively showing four main clusters (Figure 3). The results of the AMOVA analysis showed that most of the genetic variation was within the populations, while 11% genetic variation components existed among populations. Similar observations have reported for *D. oliveri* (12.6%) [54] and *D. sissoo* (14.6%) [59], which may be due to the fact that woody species with a predominately outcrossing tend to have less differentiation among populations and high variation within populations [60].

### 4.2. The Core Collection of D. odorifera Germplasm

Core collections are subsamples of large germplasm collections that include the highest genetic diversity with the minimum number of representative accessions [19]; the development of a core collection in a manageable sized subset will largely reduce redundant labors in the limited breeding cycles and making significant advances in genetic improvement. Therefore, based on 19 neutral selectively microsatellite markers (Table S3), we developed an efficient core collection using the PowerCore software with the advanced M-strategy, associating with the neighbor joining (NJ) analysis and Unweighted Pair Group Method with Arithmetic Mean (UPGMA) analysis based on the matrixes of genetic dissimilarity and Nei’s unbiased genetic distance, respectively [15,20,24,35,61,62]. The core collection comprised 31 individuals sufficient to retain all the alleles identified from the *D. odorifera* database (Figure 4; Table S1), of which, the total alleles and observed number of alleles was 77 and 4.05, respectively, exactly the same as the whole database. Thus, it guaranteed the preservation of alleles, which is essential for maintaining the genetic diversity of a population [63]. Moreover, the observed and expected heterozygosity (Ho and He) values calculated on the core collection were 0.33 and 0.44, respectively, showing no significant difference to the whole database, neither did the other genetic diversity indices (Table 5). In the core collection, the observed heterozygosity of the five populations varied from 0.25 (RP3) to 0.40 (RP1), the expected heterozygosity varied from 0.36 (RP2) to 0.44 (RP4) (Table S5), and the values of pairwise Fst (genetic differentiation among populations) from 0.052, between MIX and RP2, to 0.170, between RP3 and RP2 (Table S6). These findings demonstrated that the core collection represented sufficient genetic variation of the whole database. Additionally, the members of the core collection distributed evenly among the 251 *D. odorifera* individuals were validated by the results of the NJ and UPGMA analysis (Figure 4 and Figure 5).

As a whole, the 31 genotypes selected for the core collection are representative samples of the diversity retained from the whole southern China that covering its native habitat and the whole first introduced sites. Notably, the wild resource of *D. odorifera* is highly endangered and rare due to the overexploitation, and their distributions in their original habitat (Hainan Island) are severely fragmented but relationships among individuals are badly influenced by human activities. It is no wonder then that the core collection comprised only six wild individuals compared to 25 cultivated ones.

Prior to the present study, no information has been reported on core collection of *D. odorifera*. Therefore, this core collection can serve a basic reference on similar research for *D. odorifera* and other *Dalbergia* species. Furthermore, it can be considered as a powerful tool for exploring the genetic diversity, as well as a new source for efficient conservation and breeding programs of *D. odorifera* in the future.

## 5. Conclusions

The present study provides an overall assessment on genetic diversity and structure of 251 *D. odorifera* germplasm. A medium level of genetic diversity and genetic variation was presented within the species. Based on the assessment, a core collection was first established using the PowerCore software with the M-strategy. The core collection contained 12.4% (31) out of the 251 germplasm, possessing the intact genetic diversity of the whole germplasm collection. This core collection will serve as a primary source for further genetic association, functional analyses, and function to improve breeding programs in future studies.

## Figures and Tables

**Table 1:** Diversity statistics of the 19 microsatellite markers across *Dalbergia odorifera* germplasm.

Locus	Size	Na	Ne	Ho	He	GD	PIC	I	Nm	F	FNA
S01	251	4	2	0.46	0.50	0.5	0.43	0.87	1.93	0.08	0.03 ^no^
S02	251	3	1.06	0.05	0.06	0.06	0.06	0.15	5.63	0.11	0.0 ^no^
S03	249	3	1.98	0.38	0.50	0.49	0.37	0.70	2.53	0.23	0.08 *
S04	251	4	1.64	0.26	0.39	0.39	0.36	0.72	0.44	0.35	0.1 *
S07	251	2	1.87	0.44	0.47	0.47	0.36	0.66	3.22	0.05	0.02 ^no^
S08	251	4	1.46	0.29	0.31	0.31	0.29	0.62	5.67	0.06	0.01 ^no^
S09	251	4	2.39	0.45	0.58	0.58	0.53	1.08	2.60	0.23	0.08 *
S10	250	4	1.92	0.46	0.48	0.48	0.42	0.83	3.37	0.04	0.01 ^no^
S11	251	2	1.62	0.36	0.38	0.38	0.31	0.57	2.49	0.05	0.01 ^no^
S12	251	2	1.42	0.25	0.30	0.30	0.25	0.47	2.73	0.15	0.04 ^no^
S21	250	7	3.48	0.69	0.71	0.71	0.66	1.34	2.45	0.03	0.01 ^no^
S22	251	6	1.28	0.19	0.22	0.22	0.21	0.53	9.45	0.13	0.02 *
S23	251	6	1.04	0.02	0.04	0.04	0.04	0.12	33.31	0.33	0.01 *
S24	251	6	2.33	0.49	0.57	0.57	0.50	0.99	1.23	0.15	0.05 *
S26	251	3	1.03	0.02	0.03	0.03	0.03	0.08	9.53	0.28	0.01 *
S27	251	4	1.3	0.21	0.23	0.23	0.20	0.41	6.58	0.08	0.01 ^no^
S28	251	2	1.7	0.29	0.41	0.41	0.33	0.6	2.18	0.29	0.08 *
S29	251	6	1.91	0.44	0.48	0.48	0.39	0.78	1.07	0.07	0.02 ^no^
S30	251	5	1.92	0.43	0.48	0.48	0.40	0.79	1.68	0.11	0.04 ^no^
Mean	251	4.05	1.76	0.33	0.38	0.38	0.32	0.65	5.16	0.15	-

Size number of individuals with successful amplicons; Na: observed number of alleles; Ne: effective number of alleles; Ho: observed heterozygosity: He: expected heterozygosity; GD: Nei’s gene diversity; PIC: polymorphic information content; I: Shannon’s Information index; Nm: gene flow, estimated from Fst, Nm = [(1/Fst)—1]/4; F: fixation index; FNA: frequency of null alleles: ^no^ contained no null allele, * likely contained null alleles (*p* < 0.05).

**Table 2:** Summary on genetic diversity statistics of *Dalbergia odorifera* populations.

Population	Size	Wild	Alleles	Na	Ne	Np	Ho	He	GD	PPB %
MIX	86	13	66	3.47	1.74	9	0.34	0.36	0.36	100.00
RP1	32	5	48	2.53	1.63	2	0.34	0.33	0.32	100.00
RP2	51	3	48	2.53	1.62	1	0.33	0.32	0.32	94.74
RP3	41	15	53	2.79	1.55	2	0.27	0.30	0.30	89.47
RP4	41	6	51	2.68	1.84	3	0.33	0.38	0.37	94.74
Mean	50.2	8.4	53.2	2.80	1.68	-	0.32	0.34	0.33	95.79
Total ^a^	251	42	77	4.05	1.76	-	0.33	0.38	0.38	100

Population see Figure 2 and Table S1; Size: number of individuals; Wild: number of wild individuals; Alleles: total number of detected alleles; Na: observed mean number of alleles; Ne: mean effective number of alleles; Np: number of private alleles; Ho: observed heterozygosity; He: expected heterozygosity; GD: Nei’s gene diversity; PPB %: the percentage of polymorphic loci; a diversity indices averaged over the 19 loci across all *D. odorifera* individuals, b total number of individuals.

**Table 3:** Analysis of molecular variance (AMOVA) for *Dalbergia odorifera* populations.

Source	*d.f.*	Sum of Square	Mean of Square	Variance Components	Percentage of Variation
Among populations	4	171.788	42.947	0.408	11% ***
Within populations	497	1621.023	6.527	3.264	89%
Among Individuals	246	844.523	3.433	0.170	5% ***
Within Individuals	251	776.500	3.094	3.094	84%
Total	501	1792.811		3.671	100%

*d.f.* degrees of freedom, *** significant of data rand probability, *p* < 0.001.

**Table 4:** The matrix of pairwise genetic differentiation among populations (Fst).

Population	MIX	RP1	RP2	RP3	RP4
MIX					
RP1	0.032				
RP2	0.031	0.065			
RP3	0.041	0.091	0.095		
RP4	0.032	0.071	0.064	0.087	

**Table 5:** Comparisons on genetic diversity indices between the core collection and the whole *Dalbergia odorifera* database.

Population	Size	Na ^a^	Ne ^a^	Ho ^a^	He ^a^	GD ^a^
Core collection	31	4.05	1.96	0.34	0.44	0.44
Whole database	251	4.05	1.76	0.33	0.38	0.38

Size: number of individuals; Na: observed number of alleles; Ne: effective number of alleles; Ho: observed heterozygosity; He: expected heterozygosity; GD: Nei’s gene diversity, **^a^** no significant difference between the core collection and the whole database, the paired samples *t*-test: t 1.848, Sig. (2-tailed) 0.138, *df:* 4.

## Supplementary Materials

The following are available online at https://www.mdpi.com/2073-4425/10/4/281/s1, Figure S1: Principal coordinate analysis (PCoA) based on pairwise Nei’s unbiased genetic distance. Table S1: Information of 251 *Dalbergia odorifera* germplasm, Table S2: Details of 19 microsatellite markers, Table S3: The overall Ewens–Watterson test for neutrality across 19 microsatellite loci in *Dalbergia odorifera* germplasm, Table S4: Diversity statistics of the 19 microsatellite markers across the core collection of 30 *Dalbergia odorifera* individuals, Table S5: Summary on genetic diversity statistics of *Dalbergia odorifera* core collection, Table S6: Pairwise genetic differentiation index (Fst) values in core collection.
