# Supplementary figures and images for: Genetic Diversity and Population Structure Analysis of Dalbergia Odorifera Germplasm and Development of a Core Collection Using Microsatellite Markers
Source: Genes (Basel). 2019 Apr 6;10(4):281. doi: 10.3390/genes10040281 (PMC6523640; doi:10.3390/genes10040281)

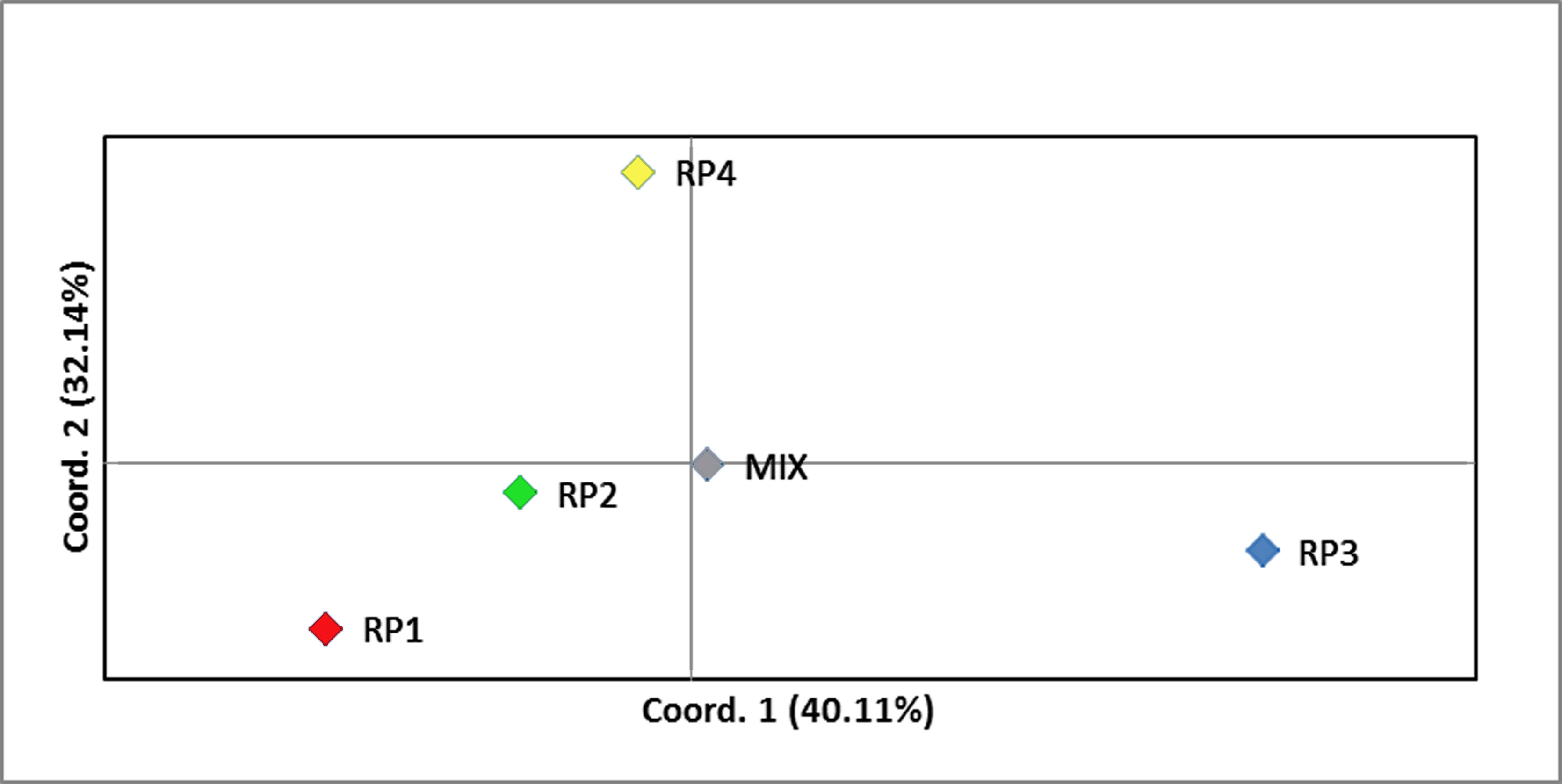

Supplement: Supplementary file 1 [file genes-10-00281-s001.zip › Figure S1 Principal coordinate analysis (PCoA) based on pairwise Neií»s unbiased genetic distance.jpg]
